# Effect of low-flow oxygen therapy on 6 minute walk test in pulmonary arterial hypertension and chronic thromboembolic pulmonary hypertension: a randomized non-inferiority crossover trial

**DOI:** 10.1093/ehjopen/oeag125

**Published:** 2026-08-25

**Authors:** Carmen Wick, Mona Lichtblau, Anna Titz, Julian Müller, Alessandro Vella, Zoe Bousraou, Michael Furian, Stéphanie Saxer, Esther I Schwarz, Silvia Ulrich

**Affiliations:** Department of Pulmonology, University Hospital Zurich, Rämistrasse 100, 8091 Zurich, Switzerland; Faculty of Medicine, University of Zurich, Rämistrasse 71, 8006 Zurich, Switzerland; Department of Pulmonology, University Hospital Zurich, Rämistrasse 100, 8091 Zurich, Switzerland; Faculty of Medicine, University of Zurich, Rämistrasse 71, 8006 Zurich, Switzerland; Department of Pulmonology, University Hospital Zurich, Rämistrasse 100, 8091 Zurich, Switzerland; Department of Pulmonology, University Hospital Zurich, Rämistrasse 100, 8091 Zurich, Switzerland; Faculty of Medicine, University of Zurich, Rämistrasse 71, 8006 Zurich, Switzerland; Department of Pulmonology, University Hospital Zurich, Rämistrasse 100, 8091 Zurich, Switzerland; Faculty of Medicine, University of Zurich, Rämistrasse 71, 8006 Zurich, Switzerland; Department of Pulmonology, University Hospital Zurich, Rämistrasse 100, 8091 Zurich, Switzerland; Faculty of Medicine, University of Zurich, Rämistrasse 71, 8006 Zurich, Switzerland; Department of Pulmonology, University Hospital Zurich, Rämistrasse 100, 8091 Zurich, Switzerland; Faculty of Medicine, University of Zurich, Rämistrasse 71, 8006 Zurich, Switzerland; Department of Pulmonology, University Hospital Zurich, Rämistrasse 100, 8091 Zurich, Switzerland; Eastern Switzerland University of Applied Sciences, Rosenbergstrasse 59, 9000 St. Gallen, Switzerland; Department of Pulmonology, University Hospital Zurich, Rämistrasse 100, 8091 Zurich, Switzerland; Faculty of Medicine, University of Zurich, Rämistrasse 71, 8006 Zurich, Switzerland; Department of Pulmonology, University Hospital Zurich, Rämistrasse 100, 8091 Zurich, Switzerland; Faculty of Medicine, University of Zurich, Rämistrasse 71, 8006 Zurich, Switzerland

**Keywords:** Pulmonary hypertension, Pulmonary arterial hypertension, Chronic thromboembolic pulmonary hypertension, Oxygen supplementation, Oxygen therapy 6-minute walking test, Supplemental oxygen therapy

## Abstract

**Aims:**

Patients with pulmonary arterial and distal chronic thromboembolic pulmonary hypertension (PAH/CTEPH) frequently experience exertional dyspnoea with desaturation. Although ambulatory low-flow supplemental oxygen therapy (SOT) is commonly prescribed, its effect via portable concentrators in daily life has not been studied. We investigated the effect of ambulatory SOT on six-minute walk distance (6MWD).

**Methods and results:**

This was a randomized non-inferiority crossover study in patients with PAH or CTEPH desaturating by >3% during exercise. Patients performed two 6MWD tests in randomized order: one in ambient air and one with pulsed low-flow (1–2 L/min) nasal supplemental oxygen (SOT) by a portable back-packed concentrator. The primary outcome was 6MWD with a predefined non-inferiority margin of −35 m for ambient air vs. SOT. Forty patients (age 62 ± 15 years, 21 female, 20 PAH, 20 CTEPH) were included. The mean difference in 6MWD between ambient air and SOT was 0 m (95% CI: −15 to 14 m, *P* <0.001).Five patients exceeded the minimal clinically important difference of 35 m with SOT. At end-exercise, the cohort desaturated 2% less with SOT (95% CI −3 to −0.2%, *P* = 0.025) and reported 1 point less dyspnoea (95% CI 0.3 to 1, *P* < 0.002). These effects were also observed in PAH but not in CTEPH.

**Conclusion:**

Walking on ambient air was non-inferior to low-flow SOT during 6MWD in treated PAH/CTEPH patients with exercise-induced desaturation and relatively preserved exercise capacity. SOT reduced dyspnoea in PAH. Future research should identify SOT responders and develop lighter, more effective devices tailored to patient needs.

**Registration:**

ClinicalTrials.gov (NCT06384534)

## Background

Pulmonary hypertension (PH) is a complex, progressive disorder affecting approximately 1% of the global population,^1,2^ defined by a mean pulmonary arterial pressure >20 mmHg measured via right heart catheterization. The World Health Organization (WHO) classifies PH into five clinical groups, of which this study focused on pulmonary arterial hypertension (PAH, Group 1) and chronic thromboembolic pulmonary hypertension (CTEPH, Group 4).^3,4^

PAH/CTEPH patients commonly experience exertional dyspnoea, exercise-induced oxygen desaturation, and reduced physical performance, all of which are linked to impaired quality of life and survival.^3^ Because physical activity supports autonomy, social participation, and survival,^5,6^ interventions that preserve or improve exercise capacity are clinically important.

Supplemental oxygen therapy (SOT) is recommended in patients with PH and resting hypoxaemia (PaO_2_ < 8 kPa [≈60 mmHg]) confirmed on at least two occasions.^3,7^ However, these recommendations are largely extrapolated from studies in COPD, and there are no data demonstrating that long-term oxygen therapy alters disease progression or improves survival in PAH/CTEPH. Furthermore, evidence-based recommendations for patients with PAH/CTEPH who desaturate exclusively during exercise are lacking. Current practice is therefore largely based on expert opinion suggesting that ambulatory oxygen may be considered in patients with symptomatic benefit and correctable exercise-induced desaturation.^3^

Placebo-controlled randomized controlled trials (RCT) in PAH/CTEPH have shown that high-dose SOT (FiO_2_ 0.5) improves exercise performance during incremental and constant work-rate cycling^8,9^ and enhances metabolic and haemodynamic responses on cardiopulmonary exercise testing and right heart catheterization.^10–12^ However, delivering mobile high oxygen concentrations is challenging, as available systems are heavy, require tight-fitting masks to counteract mouth breathing, and are impractical for everyday use.^13,14^

Evidence for commonly prescribed low-flow ambulatory SOT via nasal prongs is limited, with mixed results in chronic obstructive pulmonary disease and fibrotic interstitial lung disease.^15–18^ Factors such as delivery mode, mouth breathing during exertion, oxygen dose, and device weight likely influence effectiveness. Notably, the effects of pulsed, low-dose ambulatory SOT from portable concentrators have not yet been studied in patients with pulmonary vascular disease.

Given the uncertain evidence, and considering the logistical and financial implications of SOT, the clinically relevant research question is whether patients can maintain exercise performance without use of low-flow ambulatory oxygen. Therefore, this study was designed as a non-inferiority trial to determine whether walking on ambient air results in a clinically similar 6-minute walk distance, a validated assessment in this cohort, compared with walking using pulsed low-flow portable SOT in patients with PAH/CTEPH. The 6MWT was selected because it is a validated measure of exercise capacity and prognosis in pulmonary hypertension.^3,19^

## Methods

### Study design

This was a pragmatic randomized controlled crossover study which aimed to determine whether walking on ambient air is non-inferior to low-flow portable SOT in PAH/CTEPH in terms of 6MWD. This trial was conducted in the outpatient clinic at the University Hospital Zurich, Switzerland, between April 2024 and July 2025. A crossover design was chosen to better account for the substantial between-patient variability in 6MWD. The trial was conducted in accordance with the Declaration of Helsinki, approved by the local ethics committee (KEK Zürich, BASEC-Nr. 2024-00102) and registered at ClinicalTrials.gov (NCT06384534).

### Study population

Adults either diagnosed with PAH (idiopathic, hereditary, or associated PAH) or CTEPH^3^ were included, who revealed oxygen desaturation by pulse oximetry (SpO_2_) of >3% in the last 6MWD or cardiopulmonary exercise test, on stable PH-targeted medication at least 14 days prior to screening. Exclusion criteria were inability to comply with the study protocol due to language problems, psychological, neurological or orthopaedic disorders with inability to walk, pregnancy, or severe resting hypoxemia defined as PaO_2_ < 6.9 kPa. Screening was carried out by the study team, who reviewed the appointment lists from the PH clinic. Patients were recruited consecutively. All patients provided written informed consent.

### Study procedure, assessments, and outcomes

Two 6MWTs were conducted in randomized order, one with ambient air and one with SOT, with a washout period of minimum 1 h to maximum 1 week. No familiarization tests were performed as all participants were already familiar with the 6MWT. Tests were conducted according to American Thoracic Society guidelines.^19^ Randomization was performed using block randomization with randomly permuted blocks of four generated in R (blockrand package, version 4.5.0). The allocation sequence was created by an investigator independent of measurements and stored separately. Enrolling investigators assigned patients consecutively to pregenerated study IDs to ensure allocation concealment.

SOT was delivered using a portable oxygen concentrator (Philips SimplyGo Mini, Horgen, Switzerland, weight: 2.3 kg) carried in a backpack, provided by the device manufacturer. The device was set to level 5 for all participants, providing a 50 mL bolus of oxygen per breath. At a breathing frequency of 20 breaths per minute, this corresponds to a total oxygen delivery of about 1 l/min which increases with higher breathing frequencies during exercise.

The primary outcome was the difference in 6MWD between SOT and ambient air. Secondary outcomes included SpO_2_, blood pressure and heart rate, all measured once at the start and at the end of the exercise. Additionally, Borg-10 dyspnoea and leg-fatigue scores were rated by the patients immediately after completing the 6MWT, on a 1–10 scale, where 1 indicated no symptoms and 10 represented very severe dyspnoea or leg fatigue.^20^

After the second 6MWT, patients reported which condition they preferred and why. Baseline characteristics, including lung function and right heart catheterization data, were taken from the most recent clinical assessments. Participants remained on their usual medication regimen, with no changes in medication between the two 6MWTs.

### Statistical analysis and sample size

The sample size calculation was based on the primary outcome, 6MWD, using a non-inferiority margin of 35 m, the established clinically meaningful difference and therefore represents the largest difference considered clinically acceptable.^21,22^ Appling the method by Rothmann, Wiens and Chan^20^ with a within-subject standard deviation (SD) of 45 m, α = 0.05, β = 0.8 and an expected difference of 8 m, a sample size of 18 participants was required per diagnostic group. To account for uncertainty, it was aspired to include 20 patients per group (PAH/CTEPH), resulting in a total of 40 patients. Randomization was performed within the overall crossover design to allocate participants to one of two test sequences, ambient air followed by SOT or SOT followed by ambient air.

Continuous variables are presented as means ± SD median [IQR], as appropriate, and categorical variables in absolute and relative numbers. The primary analysis compared 6MWD between SOT and ambient air using a linear mixed-effects model in an intention-to-treat framework. Missing data were handled implicitly by the model under a missing-at-random assumption. Non-inferiority was assessed against the predefined −35 m margin, the minimal clinically important difference (MCID).^21^ Model assumptions, including homogeneity and normality of residuals, were assessed visually using Tukey-Anscombe and Q-Q plots. Potential covariates (diagnostic group, sex, age, BMI, baseline diffusion capacity (DLCO) as well as period and carry-over effect were evaluated. Exploratory analyses examined predictors of a clinically meaningful response, defined as an improvement of >35 m and, exploratively, >20 m. Secondary outcomes were analysed with the same model structure using two-sided tests. Analyses were conducted in RStudio (version 4.5.0), with statistical significance set at *P* < 0.05.

## Results

### Baseline characteristics

**Figure 1:**
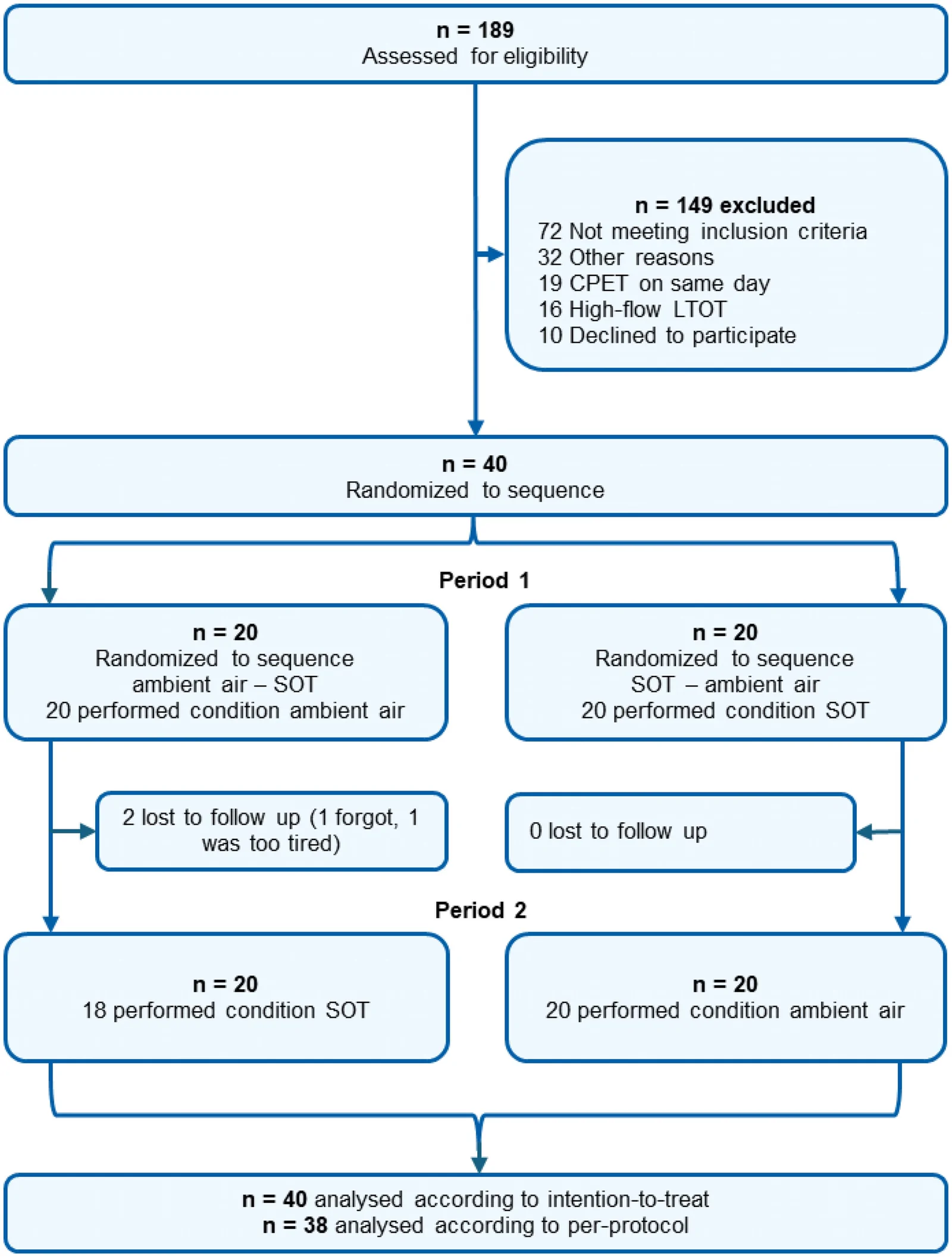
Consort flow chart for crossover studies. Abbreviations: Long-term oxygen therapy (LTOT), supplemental oxygen therapy (SOT), cardiopulmonary exercise testing (CPET).

40 patients were included, 20 with PAH and 20 with CTEPH, all under stable treatment. In the CTEPH group, two patients did not attend the second measurement (*Figure 1*). Both sexes were nearly equally represented across groups, and the distribution of age, BMI, and WHO functional class was representative of the respective populations. All baseline characteristics are listed in *Table 1*.

**Table 1:** Baseline characteristics of the PAH and CTEPH group separately and combined

Baseline characteristics	Overall	PAH	CTEPH
n	40	20	20
Male	19 (48%)	8 (40%)	11 (55%)
Female	21 (53%)	12 (60%)	9 (45%)
Age (years)	62 ± 15	61 ± 13	63 ± 18
BMI (kg/m^2^)	26.6 ± 5.2	25.3 ± 5.2	27.9 ± 4.9
WHO functional Class			
1–2	28 (70%)	11 (55%)	17 (85%)
3–4	12 (30%)	9 (45%)	3 (15%)
4-Strata risk class			
1	22 (55%)	9 (45%)	13 (65%)
2	14 (35%)	8 (40%)	6 (30%)
3	4 (10%)	3 (15%)	1 (5%)
4	0 (0%)	0 (0%)	0 (0%)
PH-targeted medication			
CCB	2 (5%)	2 (10%)	0 (0%)
ERA	21 (52.5%)	17 (85%)	4 (20%)
sGC	9 (22.5%)	3 (15%)	6 (30%)
PDE-5	15 (37.5%)	12 (60%)	3 (15%)
Sotatercept	1 (2.5%)	1 (5%)	0 (0%)
Prostacyclin infusion therapy (s.c./iv)	5 (12.5%)	5 (25%)	0 (0%)
Selexipag	5 (12.5%)	4 (20%)	1 (5%)
None	12 (30%)	1 (5%)	11 (55%)
Mono therapy	9 (22.5%)	5 (25%)	4 (20%)
Dual therapy	8 (20%)	5 (25%)	3 (15%)
Triple therapy	7 (17.5%)	6 (30%)	1 (5%)
Quadruple therapy	4 (10%)	4 (20%)	0 (0%)
Nocturnal oxygen therapy	9 (22.5%)	6 (30%)	3 (15%)
PEA	11 (27.5%)	0 (0%)	11 (55%)
BPA	6 (15%)	0 (0%)	6 (30%)
DLCO % predicted	59 ± 19.1	49.9 ± 15.4	70.5 ± 17.3
pH	7.45 ± 0.04	7.46 ± 0.03	7.43 ± 0.04
PaO_2_ (kPa)	9.4 ± 1.7	9.5 ± 2.0	9.3 ± 1.5
PaCO_2_ (kPa)	4.5 ± 0.6	4.4 ± 0.7	4.5 ± 0.5
SpO_2_ (%)	94.1 ± 3.5	94.3 ± 3.5	94.0 ± 3.7
Hb (g/L)	144 ± 29	141 ± 20	146 ± 37
Lactate (mmol/L)	0.9 [0.7, 1.2]	1.2 [0.8, 1.6]	0.8 [0.6, 1.2]
mPAP (mmHg)	38.7 ± 10.7	41.3 ± 10.6	35.9 ± 10.2
PAWP (mmHg)	11.3 ± 2.4	11.0 ± 2.4	11.7 ± 2.3
CI (l/min/m^2^)	2.8 ± 0.7	2.9 ± 0.8	2.8 ± 0.6
PVR (WU)	4.8 [3.8, 6.8]	5.9 [4.4, 8.4]	4.1 [3.6, 5.8]

Categorical variables are given in absolute and relative numbers, continuous in mean ± SD or median and [IQR] as appropriate.

Abbreviations: BMI, Body mass index; DLCO, diffusing capacity of the lungs for carbon monoxide; PaO_2_, partial pressure of oxygen in the arterial blood; PaCO_2_, partial pressure of carbon monoxide in the arterial blood; Hb, haemoglobin; SpO_2_, oxygen saturation; mPAP, mean pulmonary arterial pressure; PAWP, pulmonary artery wedge pressure; CI, cardiac index; PVR, pulmonary vascular resistance; CCB, calcium channel blocker; ERA, endothelin receptor antagonist; sGC, soluble guanylate cyclase stimulator; PDE-5, phosphodiesterase type 5 inhibitor; combination therapy (number of patients with two or more PH-targeted medications); PEA, pulmonary endarterectomy; BPA, balloon pulmonary angioplasty; PGI_2_ analogue, Iv/sc prostacyclin analogue.

### 6-minute walking distance

**Figure 2:**
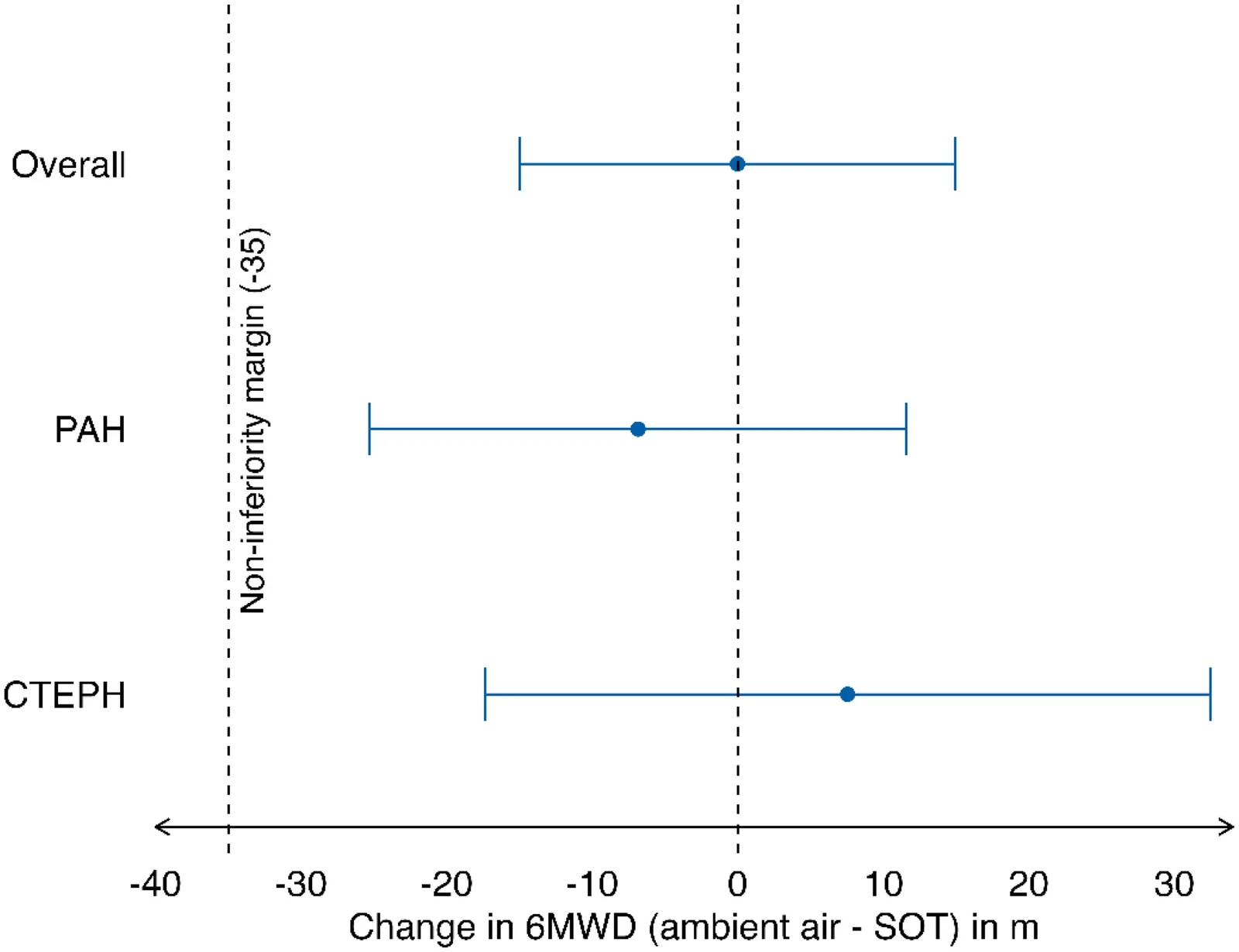
Six-minute walking distance (6MWD) overall and by diagnosis (pulmonary arterial hypertension (PAH) and chronic thromboembolic pulmonary hypertension (CTEPH) with non-inferiority margin for supplemental oxygen (SOT).

The final unadjusted linear mixed-effects model, including participant ID as random effect, demonstrated non-inferiority of ambient air compared with SOT for 6MWD (0 m (−15 to 14 m), *P* < 0.001). Non-inferiority was also demonstrated for PAH and CTEPH when analysed separately (*Table 2*, *Figure 2*).

**Table 2:** Six-minute walking distance (6MWD) outcomes combined and separate per diagnosis group

6MWD (m)	Ambient air Mean ± SD	SOT Mean ± SD	Paired mean difference (95% CI) Air-SOT	*P*-value one-sided
Overall	497 ± 120	497 ± 103	0 (−15 to 14)	**<0**.**001**
PAH	495 ± 120	502 ± 103	−7 (−24 to 10)	**<0**.**001**
CTEPH	500 ± 122	493 ± 106	7 (−16 to 31)	**<0**.**001**

Bold values indicate statistical significance at *P* < 0.05. Abbreviations: Air, ambient air; SOT, supplemental oxygen therapy.

To evaluate potential confounding, the predefined covariates, sex, age, BMI, diagnostic group, baseline DLCO, period and carryover effects were assessed within the linear mixed-effects model. Of these, only female sex and older age were statistically significantly associated with shorter 6MWD. No interaction effects between conditions (SOT vs. ambient air) and neither period nor carryover effects were detected. The inclusion of sex and age did not improve model performance; therefore, the final analysis was based on the unadjusted linear mixed-effects model with participant ID included as a random effect. A sensitivity analysis comparing the adjusted (sex and age) and unadjusted models confirmed the robustness of the results.

### Clinical characteristics as predictors of response

We identified five responders who improved their 6MWD by the minimal clinically important difference >35 m, while no participant fell into the exploratory responder range (20–35 m). The explorative descriptive analysis indicated that patients classified as responders had, on average, lower predicted DLCO, higher NYHA class, and lower 6MWD in ambient air and were more often diagnosed with PAH than CTEPH (see Supplementary material online, *Table S1*). Other variables were similar.

### Secondary outcomes

All secondary outcomes are listed in *Table 3*. Overall, patients walking with SOT experienced, on average, 2% less oxygen desaturation compared with ambient air (−2% [−3% to −0.2%] *P* = 0.025). This was also observed for the PAH group (−2% [−4 to −0.3], *P* = 0.022). In contrast, no significant difference in oxygen desaturation between SOT and ambient air was found in the CTEPH subgroup (−1% [−3% to 1%] *P* = 0.402).

**Table 3:** Secondary outcomes overall and separate per diagnosis group

Overall								
Outcome	Ambient air			SOT			Delta mean difference ambient air -SOT (95%-CI)	*P*-value two-sided *P*-value
	Rest	Peak	Paired difference (peak-rest)	Rest	Peak	Paired difference (peak-rest)		
Dyspnoea (Borg)	NA	5 ± 3	NA	NA	4 ± 3	NA	1 (0.3 to 1)	**<0**.**002**
Leg Fatigue (Borg)	NA	3 ± 3	NA	NA	3 ± 3	NA	0 (0 to 1)	0.263
SpO_2_ (%)	96 ± 3	89 ± 6	−7 ± 5	96 ± 3	91 ± 6	−5 ± 5	−2 (−3 to −0.2)	**0**.**025**
HR (bpm)	84 ± 17	118 ± 28	33 ± 23	84 ± 14	120 ± 21	36 ± 19	−3 (−9 to 3)	0.312
Systolic BP (mmHg)	126 ± 19	152 ± 32	26 ± 26	129 ± 22	154 ± 28	24 ± 25	2 (−6 to 9)	0.691
Diastolic BP (mmHg)	80 ± 13	86 ± 22	7 ± 18	81 ± 13	89 ± 17	8 ± 15	−1 (−8 to 5)	0.608
**Pulmonary arterial hypertension**								
Dyspnoea (Borg)	NA	6 ± 2	NA	NA	5 ± 3	NA	1 (0.3 to 2)	**0**.**008**
Leg Fatigue (Borg)	NA	4 ± 3	NA	NA	3 ± 2	NA	1 (0.3 to 2)	**0**.**005**
SpO_2_ (%)	95 ± 3	88 ± 7	−6 ± 6	95 ± 4	91 ± 6	−4 ± 6	−2 (−4 to −0.3)	**0**.**022**
HR (bpm)	90 ± 19	121 ± 33	30 ± 26	88 ± 15	125 ± 20	37 ± 20	−7 (−16 to3)	0.154
Systolic BP (mmHg)	122 ± 16	145 ± 36	23 ± 30	128 ± 23	150 ± 34	21 ± 29	2 (−9 to 11)	0.821
Diastolic BP (mmHg)	77 ± 14	79 ± 15	1 ± 12	80 ± 16	84 ± 16	4 ± 11	−3 (−9 to 3)	0.337
**Chronic thromboembolic pulmonary hypertension**								
Dyspnoea (Borg)	NA	3 ± 2	NA	NA	3 ± 3	NA	0 (0 to 1)	0.072
Leg Fatigue (Borg)	NA	3 ± 3	NA	NA	3 ± 3	NA	−1 (−1 to −0.04)	**0**.**034**
SpO_2_ (%)	96 ± 2	89 ± 6	−7 ± 5	98 ± 2	91 ± 5	−6 ± 5	−0.5 (−3 to 1)	0.402
HR (bpm)	77 ± 13	115 ± 23	37 ± 20	79 ± 12	113 ± 21	35 ± 19	0 (−6 to 7)	0.808
Systolic BP (mmHg)	130 ± 20	159 ± 28	28 ± 22	131 ± 22	158 ± 21	27 ± 20	2 (−11 to 13)	0.818
Diastolic BP (mmHg)	82 ± 12	93 ± 26	12 ± 21	81 ± 9	94 ± 17	12 ± 18	1 (−12 to 11)	0.921

For the rest, peak, mean and differences of the true values were taken, 95% CI and *P*-values were calculated with the model. Differences per conditions were calculated peak minus rest values. Differences between conditions were calculated ambient air minus SOT. For dyspnoea and leg fatigue instead of the delta mean differences were the paired differences calculated. Significant *P*-values are marked bold.

Abbreviations: HR, heart rate; BP, blood pressure.

Heart rate and blood pressure at rest and at peak exercise were similar with air and SOT overall and within both diagnostic groups.

Overall and within the PAH group, Borg dyspnoea scores were lower with SOT (mean difference ambient air minus SOT: 1 [0.3 to 1], *P* = 0.002 and 1 [0.3 to 2], *P* = 0.008, respectively) whereas no significant difference was observed in the CTEPH group (mean difference 1 [−0.1 to 1], *P* = 0.072). Leg fatigue showed differing trends: It decreased with SOT in the PAH group (mean difference 1 [0.3 to 2], *P* = 0.005) but increased in the CTEPH group (mean difference −1 [−1 to −0.04], *P* = 0.034), resulting in no overall difference in the combined analysis (mean difference 0 [−0.2 to 1], *P* = 0.263).

### Preference

**Figure 3:**
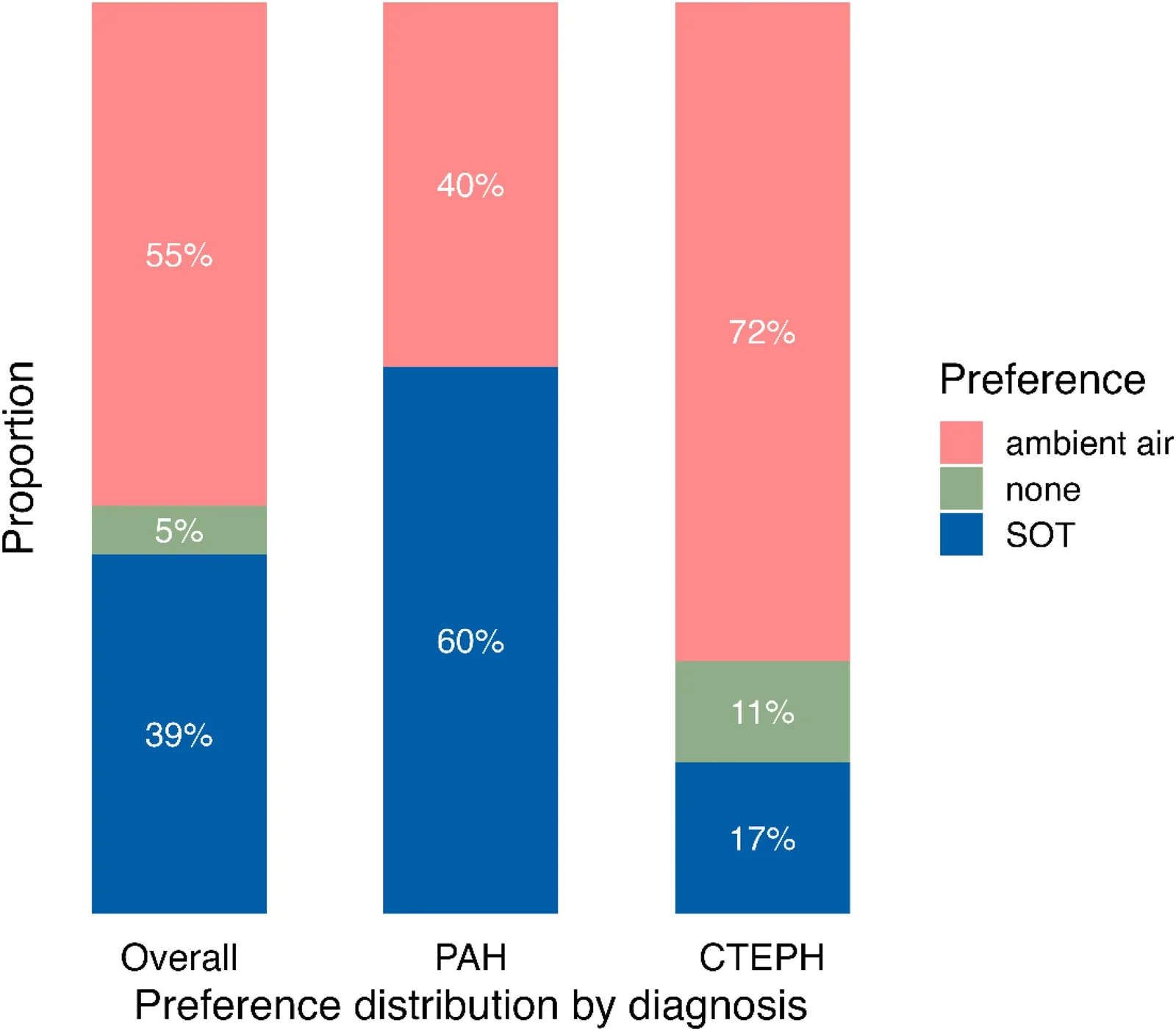
Visualization of the preference by diagnosis (pulmonary arterial hypertension (PAH) and chronic thromboembolic pulmonary hypertension (CTEPH). Supplemental oxygen therapy (SOT).

In total, 55.26% of patients preferred walking with ambient air, 39.47% preferred SOT, and 5.26% had no preference (*Figure 3*). Split by diagnosis, 40% of PAH patients preferred ambient air, and 60% preferred SOT, whereas 72% of CTEPH patients preferred ambient air, 17% SOT, and 11% preferred none. A preference for a given condition did not correspond to improved performance in that condition.

In post-test interviews, patients who preferred ambient air often reported no subjective benefit from SOT and described it as bothersome. For example, one patient stated to have ‘*no subjective benefit of oxygen*’ (ID 133), while another noted, ‘*I felt restricted by the backpack*’ (ID 127). Similarly, a third patient commented, ‘*I felt no benefit from oxygen. The backpack was bothersome*’ (ID 140).

Patients who preferred SOT reported experiencing less dyspnoea and fatigue and expressed a belief that they could have walked longer with oxygen support. One participant described, ‘*I had less strenuous breathing and think I could go further with oxygen*’ (ID 103), and another stated, ‘*I find it easier to breathe and feel less exhausted at the end of the test*’ (ID 128).

## Discussion

This pragmatic RCT reflecting real-world conditions demonstrated that in patients with PAH/CTEPH and exercise-induced desaturation, the 6MWD in ambient was non-inferior to low-dose pulsed SOT delivered via nasal cannula from a portable backpack concentrator. However, walking with SOT was associated with slightly less perceived dyspnoea in PAH (but not in CTEPH) and more than half of the patients with PAH preferred SOT over ambient air. Patient preferences differed between diagnostic groups, with PAH patients tending to favour SOT and CTEPH patients tending to favour ambient air.

Previous placebo-controlled RCTs have shown that patients with PAH or distal CTEPH on optimized drug therapy improved their exercise performance with high-dose SOT (FiO_2_ 0.5) during incremental ramp and constant work-rate cycle exercise.^8,9^ Notably, these patients benefited even more than those with COPD or chronic heart disease. High-dose SOT also significantly improved metabolic parameters during cardiopulmonary exercise testing as well as haemodynamics assessed by right heart catheterization.^10–12^ Meanwhile, nocturnal or domiciliary SOT delivered via simple nasal cannulas has demonstrated improved daytime exercise performance in PAH/CTEPH patients by alleviating sleep-disordered breathing, nocturnal hypoxaemia, and unstable oxygenation reflected by exercise-induced desaturation.^23,24^ In these studies, improvements in 6MWD measured in ambient air reached +35 m and +19 m, suggesting that sufficient preoxygenation before exercise may contribute to enhanced performance. Moreover, in another RCT where SOT was administered for more than 16 h per day over 12 weeks, 6MWD improved by +42 m compared to no SOT.^25^

In contrast to these long term and short term but high-flow SOT trials,^8,9^ the present pragmatic study using low-dose SOT via nasal cannula from a portable concentrator found the 6MWD with ambient air not to be worse than with SOT. This could be explained by insufficient oxygen delivery to the alveoli during the application, as low-flow nasal oxygen may be compromised by mouth-breathing during exertion. Additionally, the weight of the backpack concentrator might encumber exercise performance. Furthermore, the standardized 6MWT performed on a flat indoor course may underestimate the practical burden of carrying a portable concentrator during daily activities, which often involve stairs, uneven surfaces, and other environmental obstacles. While higher-flow SOT systems would be desirable for ambulation, their size and weight limit practical use in daily activities, and effective interfaces such as facial masks would likely not be accepted by patients. To our knowledge, no prior studies have evaluated low-dose ambulatory SOT in PAH or CTEPH, limiting direct comparison of our findings with existing literature.

Despite the lack of objective improvement in 6MWD, overall dyspnoea ratings on the Borg-10 scale were improved with SOT. Patient-reported experiences of this study, however, varied: Some described a subjective sense of improved walking capacity, reduced dyspnoea or exhaustion, or a feeling of more safety, even when measured 6MWD was unchanged. Others, in contrast, found carrying the device physically burdensome due to its weight, warmth, vibration, and noise. The nasal cannula itself was often reported as uncomfortable, and some patients noted difficulty or even inability to adapt their breathing pattern to nasal inhalation during exertion. These findings are consistent with a qualitative systematic review in ILD patients, which highlighted the need for lighter, easier-to-manage SOT systems to overcome similar practical barriers reported by patients, caregivers, and healthcare professionals.^26^

Taken together, these results highlight the importance of considering both objective and patient-reported outcomes when evaluating SOT in PAH/CTEPH. Although walking in ambient air was non-inferior, several PAH patients reported feeling less dyspnoeic and being more capable of exertion in SOT, suggesting that perceived benefit may not be fully captured by standard functional metrics and further parameters as cardiac effort^27^ or endurance duration measured by constant work-rate exercise tests^10^ should be considered. The heterogeneity in patient responses underscores the need for individualized assessment rather than a uniform approach to SOT. While SOT improves arterial oxygenation and acts as a pulmonary vasodilator,^28^ these physiological effects do not necessarily translate into functional or subjective gains for all patients, and a brief 6MWT alone may be insufficient to identify those who benefit.

### Responder vs. non-responder

The heterogeneous responses to SOT suggest that identifying responders may support a more individualized approach to oxygen prescription. A lower 6MWD in ambient air was associated with higher odds of response to SOT. This suggests that patients with more limited baseline exercise capacity may benefit more from SOT (see Supplementary material online, *Table S1*). Considering that the ceiling effect for the 6MWT is generally reported at 450–500 m,^29–31^ the higher baseline distance in non-responders likely limited their potential for improvement. What could be considered in the future instead of evaluating the raw distances walked is looking at a performance score (T2D) which takes potential floor and ceiling effects into account.^32^ Although exploratory, the observed associations suggest that patients with lower baseline exercise capacity may derive greater benefit from SOT. Given the small number of responders, these findings should be regarded as hypothesis-generating and require confirmation in larger studies.

### Differential response of PAH vs. CTEPH

Although the diagnostic group had no statistically significant impact on the overall response to SOT, patients with PAH tended to benefit more from SOT than those with CTEPH. This trend was also reflected in patient preferences with PAH patients tending to prefer SOT and CTEPH patients tending to prefer ambient air. Importantly, no association was found between 6MWD and preference, indicating that subjective experience was not necessarily linked to objective performance.

A potential explanation for these relatively minor differences between PAH and CTEPH may be the underlying pathophysiology. In both conditions, hypoxaemia can result from low cardiac output, ventilation-perfusion (V/Q) mismatch and impaired gas exchange. However, while impaired diffusion capacity due to small-vessel remodelling may be more relevant in PAH,^4,8^ V/Q mismatch and dead-space ventilation are thought to predominate in CTEPH.^33,34^ As oxygen delivery to non-perfused lung regions is unlikely to substantially improve gas exchange, supplemental oxygen may theoretically have a smaller effect in CTEPH. However, distal forms of CTEPH often exhibit microvascular remodelling and may therefore behave similarly to PAH.^35,36^

### Limitations

This study was intentionally conducted without blinding to pragmatically assess real-world application. However, the carry-on weight of the concentrator and patients’ awareness of receiving SOT vs. ambient air may have introduced bias, particularly in subjective outcomes such as dyspnoea, leg fatigue, and patient preference, as well as in the objective 6MWD due to variations in effort. Furthermore, the oxygen delivery provided by the portable concentrator was estimated based on device specifications and likely varied with breathing frequency during exercise. Therefore, the exact amount of oxygen delivered to individual participants was not quantified. The short duration of SOT during the 6MWT does not permit conclusions regarding longer-term, nocturnal, or domiciliary oxygen therapy and its potential benefits. Additionally, the relatively high baseline 6MWD of the study population may limit the generalizability of these findings to more severely impaired patients with lower exercise capacity.

## Conclusion

In this pragmatic non-inferiority trial in treated PAH or CTEPH patients with exercise-induced hypoxaemia and relatively preserved exercise capacity, walking with ambient air resulted in a similar 6MWD as with pulsed low-flow nasal SOT delivered by a portable concentrator. The lack of a performance advantage may be attributable to insufficient oxygen delivery combined with the additional weight. Nevertheless, some PAH patients reported subjective improvement, underscoring potential value for selected individuals. The heterogeneous responses highlight the need for personalized assessment and for lighter, more effective ambulatory oxygen systems. Future research should focus on identifying responders and optimizing oxygen dose, interfaces, and portable devices.

## Supplementary Material

oeag125_Supplementary_Data

## Data Availability

The anonymized dataset and codebook will be uploaded to the data repository zenodo.
