# Supplementary material for: Effect of low-flow oxygen therapy on 6 minute walk test in pulmonary arterial hypertension and chronic thromboembolic pulmonary hypertension: a randomized non-inferiority crossover trial
Source: Eur Heart J Open. 2026 Aug 25;6(5):oeag125. doi: 10.1093/ehjopen/oeag125 (PMC13537019; doi:10.1093/ehjopen/oeag125)
Supplement: Supplementary file 1 [file oeag125_supplementary_data.docx]

**Supplementary Material**

**Responder vs. Non-Responder Characteristics**

| **Table S1** |  |  |
| --- | --- | --- |
|  | **Clinically significant responder** | **Non-Responder** |
| **Baseline characteristics** |  |  |
| n | 5 | 33 |
| Male | 4 (80 %) | 14 (42 %) |
| Female | 1 (20 %) | 19 (58 %) |
| Age (years) | 62 ± 11 | 62 ± 16 |
| BMI (kg/m^2^) | 27.1 ± 4.1 | 26.3 ± 5.3 |
| NYHA Class |  |  |
| 1-2 | 0 (0 %) | 27 (82 %) |
| 3-4 | 5 (100 %) | 6 (18 %) |
| Diagnosis |  |  |
| PAH | 4 (80 %) | 16 (48 %) |
| CTEPH | 1 (20 %) | 17 (52 %) |
| Nocturnal oxygen therapy | 1 (20 %) | 8 (24%) |
| DLCO predicted (%) | 43 ± 13 | 60 ± 18 |
| mPAP | 35 ± 11 | 40 ± 11 |
| PVR | 3.96 ± 1.83 | 6.00 ± 3.02 |
| CI | 3.43 ± 0.53 | 2.78 ± 0.72 |
| PAWP | 11.00 ± 2.16 | 11.33 ± 2.45 |
| pH | 7.47 ± 0.06 | 7.45 ± 0.04 |
| PaO_2_ (kPa) | 10.47 ± 2.37 | 9.21 ± 1.62 |
| PaCO_2_ (kPa) | 3.98 ± 0.71 | 4.54 ± 0.57 |
| SpO_2_ (%) | 96 ± 4 | 94 ± 3 |
| Hb (g/l) | 148 ± 18 | 147 ± 21 |
| Lactate (mmol/l) | 2.00 ± 0.83 | 1.01 ± 0.63 |
| **6MWT characteristics (for paired outcomes, mean paired difference are calculated)** | | |
| 6MWD ambient air | 330 ± 138 | 532 ± 99 |
| 6MWD SOT | 417 ± 88 | 510 ± 101 |
| 6MWD difference (ambient – SOT) | -87 ± 58 | 13 ± 26 |
| Heart rate ambient air (peak-rest) | 26 ± 28 | 34 ± 23 |
| Heart rate SOT (peak-rest) | 30 ± 25 | 37 ±18 |
| Systolic BP ambient air (peak-rest) | -7 ± 20 | 31 ± 24 |
| Systolic BP SOT (peak-rest) | 7 ± 40 | 27 ± 21 |
| Diastolic BP ambient air (peak-rest) | -4 ± 7 | 8 ± 19 |
| Diastolic BP SOT (peak-rest) | 8 ± 34 | 8 ± 11 |
| SpO_2_ ambient air (peak-rest) | -5 ± 5 | -7 ± 6 |
| SpO_2_ SOT (peak-rest) | -4 ± 7 | -5 ± 5 |
| Dyspnea ambient air (peak-rest) | 5 ± 2 | 4 ± 3 |
| Dyspnea SOT (peak-rest) | 5 ± 3 | 4 ± 3 |
| Leg fatigue ambient air (peak-rest) | 6 ± 3 | 3 ± 3 |
| Leg fatigue SOT (peak-rest) | 6 ± 3 | 3 ± 3 |

Table S1: Characteristics of responder vs. non-responders. As responder were classified patients walking ≥ 35 m further with SOT. Only patients with completion of both 6MWT were included. Abbreviations: Body mass index (BMI), diffusing capacity of the lungs for carbon monoxide (DLCO), partial pressure of oxygen in the arterial blood (PaO2), partial pressure of carbon monoxide in the arterial blood (PaCO2), hemoglobin (Hb), oxygen saturation (SpO2), mean pulmonary arterial pressure (mPAP), pulmonary artery wedge pressure (PAWP), cardiac index (CI), pulmonary vascular resistance (PVR), blood pressure (BP).
